# Hydrazinobenzoylcurcumin inhibits androgen receptor activity and growth of castration-resistant prostate cancer in mice

**DOI:** 10.18632/oncotarget.3346

**Published:** 2015-01-31

**Authors:** Min Wu, Sahn-Ho Kim, Indrani Datta, Albert Levin, Gregory Dyson, Jing Li, Stephanie Kaypee, M. Mahadeva Swamy, Nilesh Gupta, Ho Jeong Kwon, Mani Menon, Tapas K. Kundu, G. Prem-Veer Reddy

**Affiliations:** ^1^ Vattikuti Urology Institute, Henry Ford Hospital, Detroit, MI, USA; ^2^ Bioinformatics Core, Public Health Sciences, Henry Ford Hospital, Detroit, MI, USA; ^3^ Biostatistics Core, Karmanos Cancer Institute, Wayne State University, Detroit, MI, USA; ^4^ Pharmacology Core, Karmanos Cancer Institute, Wayne State University, Detroit, MI, USA; ^5^ Transcription and Disease Laboratory, Molecular Biology and Genetics Unit, JNCASR, Bangalore, Karnataka, India; ^6^ Department of Pathology, Henry Ford Hospital, Detroit, MI, USA; ^7^ Department of Biotechnology, Translational Research Center for Protein Function Control, Yonsei University, Seoul, Republic of Korea

**Keywords:** Androgen receptor, calmodulin, Hydrazinobenzoylcurcumin, CTK7A, castration-resistant prostate cancer

## Abstract

There is a critical need for therapeutic agents that can target the amino-terminal domain (NTD) of androgen receptor (AR) for the treatment of castration-resistant prostate cancer (CRPC). Calmodulin (CaM) binds to the AR NTD and regulates AR activity. We discovered that Hydrazinobenzoylcurcumin (HBC), which binds exclusively to CaM, inhibited AR activity. HBC abrogated AR interaction with CaM, suppressed phosphorylation of AR Serine81, and blocked the binding of AR to androgen-response elements. RNA-Seq analysis identified 57 androgen-regulated genes whose expression was significantly (p ≤ 0.002) altered in HBC treated cells as compared to controls. Oncomine analysis revealed that genes repressed by HBC are those that are usually overexpressed in prostate cancer (PCa) and genes stimulated by HBC are those that are often down-regulated in PCa, suggesting a reversing effect of HBC on androgen-regulated gene expression associated with PCa. Ingenuity Pathway Analysis revealed a role of HBC affected genes in cellular functions associated with proliferation and survival. HBC was readily absorbed into the systemic circulation and inhibited the growth of xenografted CRPC tumors in nude mice. These observations demonstrate that HBC inhibits AR activity by targeting the AR NTD and suggest potential usefulness of HBC for effective treatment of CRPC.

## INTRODUCTION

Prostate cancer (PCa) is the most frequently diagnosed non-skin cancer and second leading cause of cancer related deaths in American men [1]. Androgen, by activating the androgen receptor (AR), plays a pivotal role in PCa cell proliferation and viability [2]. Hence, androgen-deprivation is frontline therapy for the treatment of disseminated PCa. However, this treatment does not provide a lasting remission as the disease invariably recurs and progresses to become lethal [3]. This recurring disease, referred to as castration-resistant prostate cancer (CRPC), continues to depend on AR signaling, and becomes resistant to all currently available forms of androgen-deprivation and AR-antagonistic therapies, which are designed to target primarily the AR ligand-binding domain (LBD) [4-6]. Therefore, there is a critical need for AR-antagonists that can inhibit AR activity through an LBD-independent mechanism for the treatment of CRPC [7].

AR belongs to the steroid receptor family of transcription factors containing four distinct functional domains; amino-terminal domain (NTD) containing activation function (AF-1), DNA binding domain (DBD), hinge region (HR) containing nuclear localization sequence, and carboxy-terminal LBD. The AR NTD is crucial for AR transcriptional activity; while AR devoid of LBD can be constitutively active, there can be no transcriptional activity without NTD [8]. A number of proteins interact with NTD and play an important role in regulation of AR activity [9]. Calmodulin (CaM) is one such co-regulator that binds to the AR NTD and regulates AR activity; CaM-antagonists, including CaM-siRNA inhibit AR activity and suppress AR protein stability in PCa cells [10-12]. Thus, targeting CaM may offer a viable strategy to inhibit AR activity through a mechanism that does not involve intact AR LBD.

CaM is a ubiquitous calcium-binding protein that has no intrinsic enzymatic activity but binds to and regulates the activity of a variety of enzymes involved in cellular processes such as cell proliferation and viability, and cancer [13]. CaM is overexpressed in a variety of cancers including liver, lung and breast [14-16]. Interestingly, CaM overexpression in breast tumor tissues is associated with increased estrogen receptor (ER) levels [17]. As in the case of AR, CaM also binds to ER and regulates its activity [18, 19]. Recently, a novel Curcumin derivative, 4-{3,5-Bis-[2-(4-hydroxy-3-methoxy-phenyl)-ethyl]-4,5-dihydro-pyra­zol-1-yl}-benzoic acid (hydrazinobenzoylcurcumin, HBC) is developed that is shown to bind exclusively to CaM and antagonize its function [20]. HBC inhibits proliferation of HCT15 colon carcinoma [20] and suppresses angiogenic activity of human umbilical vascular endothelial cells [21]. These anti-proliferative and anti-angiogenic effects of HBC are mediated by its inhibitory effect on CaM-regulated signaling pathways [20, 21]. A water-soluble form of HBC, the sodium salt of HBC, NaHBC (also known as CTK7A), was shown to inhibit the proliferation of KB oral squamous cell carcinoma cells and suppress the growth of oral tumor xenografts in mice [22]. This anti-tumorigenic effect of HBC was shown to be associated with its inhibitory effect on p300 histone acetyltransferase activity, which is mediated by nitric oxide synthase [22]; importantly, nitric oxide synthase is a CaM-regulated enzyme [23].

Since CaM regulates AR activity [10, 12], and HBC binds exclusively to CaM [20], we investigated the effect of HBC on AR activity and on the androgen-regulated transcriptome in PCa cells. In addition, we evaluated the efficacy of HBC in suppressing the growth of tumors derived from metastatic CRPC cells in mice. We report that HBC inhibits AR activity by disrupting AR-CaM interaction, reverses the expression of androgen-regulated genes associated with PCa development and progression, and suppresses the growth of CRPC tumors in mice. These observations suggest AR-antagonistic effect of HBC through a mechanism that does not involve AR LBD, and suggest potential usefulness of HBC for an effective treatment of CRPC.

## RESULTS

### CaM is over-expressed in human prostate tumor tissues

**Figure 1:**
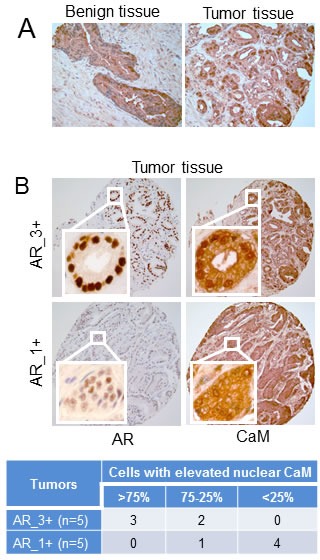
Immunohistochemistry of CaM and AR in human prostate tumor and non-tumor tissues A) Evaluation of CaM expression in non-tumor (benign) and tumor prostate tissues. B) Evaluation of CaM expression in serial sections of prostate tumors with high (AR_3+) and low (AR_1+) AR levels. Immunostaining is as described in Materials and Methods. Images are representative of five each of benign and, AR_3+ and AR_1+ tumor tissue specimens. Table shows percentage of luminal epithelial cells in AR_3+ and AR_1+ tumors with elevated nuclear CaM. Percentage of cells with elevated nuclear CaM was determined by counting more than 300 cells in each tumor tissue.

We have shown previously that CaM levels are 2- to 3- fold higher in AR-positive LNCaP cells than in AR-negative PC-3 cells [10]. We now evaluated CaM levels in human prostate tumor tissues and found a higher level and increased nuclear localization of CaM in prostate tumor tissues as compared to non-tumor (benign) prostate tissues (Fig. 1A). We tested AR and CaM expression in serial sections of tumor tissues and found a strikingly a greater nuclear accumulation of CaM in tumors that had a high level of AR than in tumors with a low AR level (Fig. 1B). As shown in the Table (Fig. 1B lower panel), 3 out of 5 AR_3+ tumors had more than 75% of luminal epithelial cells with elevated nuclear CaM. By comparison, 4 out of 5 AR_1+ tumors had less than 25% of cells with elevated nuclear CaM. These observations raise an intriguing possibility that overexpressed CaM in the nuclei of prostate tumors may facilitate AR protein stability and function.

### Differential effect of HBC on proliferation of AR-positive vs. AR-negative PCa cells

**Figure 2:**
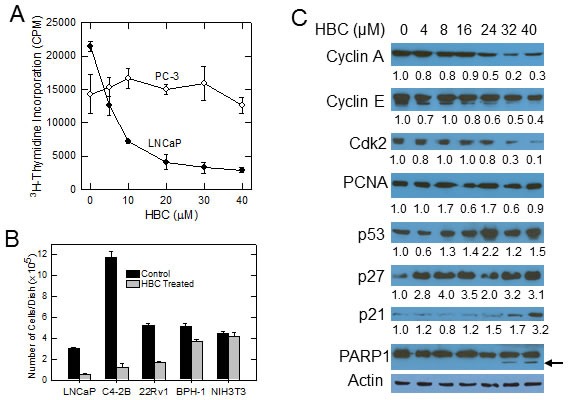
HBC inhibits proliferation of AR-positive prostate cancer cells A) Exponentially growing LNCaP and PC-3 cells were treated with increasing concentrations of HBC for 24 hours and the incorporation of pulse labeled ^3^H-thymidine into DNA was determined as described in Materials and Methods. Each data point is mean and SE obtained from 3 biological replicates, and representative of 2 independent experiments. B) Individual cell lines were plated at a density of 5×10^4^ cells/35 mm^2^ dish, treated with solvent alone (control) or 20 μM HBC starting a day after plating, and the number cells of cells in each dish was determined at day 6 as described in Materials and Methods. Each column is mean and SE of values obtained from triplicate dishes, and the data is representative of 2 independent experiments. C) Exponentially growing LNCaP cells were treated with increasing concentrations of HBC for 24 hours; cell extracts were prepared and Western blot analysis was performed as described in Materials and Methods. Band densities were determined by using the ImageJ program and β-actin-normalized relative densities are presented below each blot.

Since AR plays a critical role in proliferation and survival of PCa cells and CaM regulates AR activity [10], we tested the effect of HBC on proliferation of prostate cancer cells that are AR-positive and depend on AR for proliferation (LNCaP cells). Treatment of exponentially growing LNCaP cells with increasing concentrations of HBC resulted in a dose-dependent decrease in the incorporation of ^3^H-thymidine into DNA, indicating an anti-proliferative effect of HBC on LNCaP cells with an IC_50_ of ~5 μM (Fig. 2A). Interestingly, a similar treatment with HBC had no noticeable effect on proliferation of AR-negative PC-3 cells even at 40 μM concentration (Fig. 2A). In addition to LNCaP cells, HBC at 20 μM also inhibited proliferation of androgen-independent (castration-resistant) C4-2B and 22Rv1 cells that depend on AR for proliferation (Fig. 2B). By comparison, HBC had a marginal effect on proliferation of benign prostatic epithelial BPH-1 cells that lack AR and had a negligible effect on NIH3T3 mouse embryo fibroblast cells. Proliferation of BPH-1 and NIH3T3 cells was sensitive to HBC at higher concentrations ranging from 75 – 150 μM (data not shown), suggesting that the proliferation of AR-positive androgen-sensitive (LNCaP), as well as castration-resistant (C4-2B and 22Rv1), PCa cells exhibit a greater sensitivity to HBC than do AR-negative cells.

The inhibitory effect of HBC on LNCaP cell proliferation was associated with a corresponding decrease in cell cycle regulatory proteins, such as cyclin A, cyclin E and Cdk2, that play an important role in progression of cells from G_1_ to S phase (Fig. 2C). HBC also induced the expression of p53, p27^Kip1^, and p21^Cip1^ that negatively regulate cell cycle progression (Fig. 2C). By comparison, HBC had no noticeable effect on proliferating cell nuclear antigen (PCNA), a protein associated with DNA replication, In addition to its effect on cell cycle regulatory proteins, at higher concentrations, HBC caused PARP1 cleavage (Fig. 2C, arrow), which is a sign of apoptosis. Although there was no apparent cell death, as determined by trypan blue exclusion, there was a small increase in the rounding of cells over a 24 period (Supplementary Fig. S1), suggesting that at concentrations exceeding 40 μM, HBC may induce apoptotic cell death.

### HBC inhibits AR transcriptional activity

**Figure 3:**
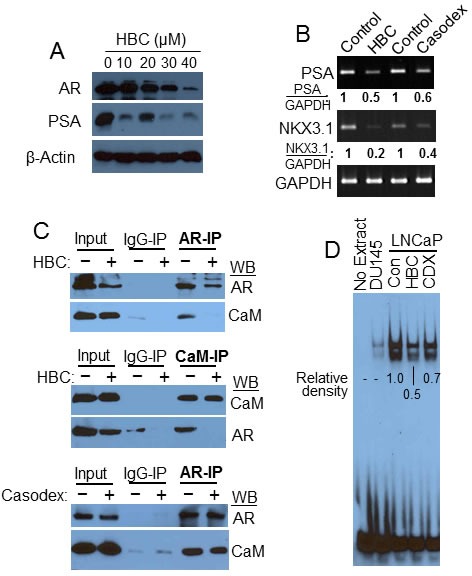
HBC inhibits AR transcriptional activity A) Exponentially growing LNCaP cells were treated with increasing concentrations of HBC for 24 hours, and AR, PSA and β-actin levels in cell extracts were determined. B) Exponentially growing LNCaP cells were treated with 40 μM HBC or Casodex for 24 hours and RT-PCR performed to evaluate PSA, NKX3.1 and GAPDH mRNA expression. The relative density of GAPDH normalized PSA and NKX3.1 bands was determined using EagleSight software (version 3.2; Stratagene). C) HBC disrupts AR-CaM interaction. Exponentially growing LNCaP cells were treated with 40 μM HBC or Casodex for 24 hours, AR and CaM immunoprecipitates were prepared and Western blot analysis was performed as described in Materials and Methods. Input represents 10% of the protein used for immunoprecipitation. Observations are representative of 3 independent experiments. D) HBC inhibits AR binding to androgen response elements. EMSA reactions were carried out in the presence of 40 μM HBC or Casodex (CDX), or vehicle (control) and subjected to non-denaturing gel electrophoresis. EMSA was performed as described in Materials and Method. The data is representative of 2 independent experiments. Density of bands in each lane were determined by using the ImageJ program and relative densities were calculated after subtracting the background density of bands in DU145 lane from the density of bands in LNCaP lanes.

Since CaM binds to AR and regulates AR protein function and stability [10, 11], we tested the effect of HBC on AR in LNCaP cells. HBC caused a noticeable decrease in AR protein levels, particularly, at 30 to 40 μM concentration (Fig. 3A). Importantly, a much lower concentration of HBC was needed to decrease the level of prostate specific antigen (PSA), an AR-target gene, than to decrease the AR protein level (Fig. 3A), indicating that the decrease in PSA expression is not due to a decrease in AR protein level and is likely due to the inhibitory effect of HBC on AR transcriptional activity. The effect on PSA instead associated with a 2-fold decrease in PSA mRNA levels (Fig. 3B). The expression of another AR target gene, NKX3.1, was decreased by 5-fold in these cells (Fig. 3B). Notably, the inhibitory effect of HBC on the expression of PSA and NKX3.1 was similar to that seen with Casodex (bicalutamide) (Fig. 3B), which binds directly to the LBD of AR and inhibits AR activity. Thus HBC inhibits AR transcriptional activity as effectively as Casodex in prostate cancer cells.

In an attempt to identify molecular events contributing to HBC-induced suppression of AR activity, we studied the effect of HBC on AR-CaM interaction. As shown in Fig. 3C, AR-immunoprecipitate (AR-IP) prepared from control untreated LNCaP cells contained CaM, indicating AR interaction with CaM as described previously [10, 11], and this interaction between AR and CaM was blocked in cells treated with HBC. Similarly, in a reciprocal analysis, CaM-IP prepared from control cells contained AR, and HBC disrupted this association (Fig. 3C), suggesting that the inhibitory effect of HBC on AR activity may involve disruption of AR-CaM interaction. By comparison, Casodex did not disrupt AR interaction with CaM (Fig. 3C). Thus, HBC and Casodex inhibit AR transcriptional activity by mechanisms that are different from one another. In order to evaluate the effect of HBC on the assembly of AR transcription complexes in chromatin, we then used EMSA to test the effect of HBC on binding of AR to PSA-ARE. As shown in Fig. 3D, the PSA-ARE oligo bound to LNCaP nuclear extract (control) and negative controls (omitting extract or using AR-negative DU145 nuclear extract) indicate this binding was AR-dependent. HBC caused a 2-fold decrease in this binding, suggesting a disruptive effect of HBC on the assembly of AR transcription complexes. Casodex on the other hand caused <2-fold decrease in this AR-ARE interaction, suggesting that HBC is more effective than Casodex in blocking AR-ARE interaction.

### HBC inhibits androgen-stimulated activity of AR

**Figure 4:**
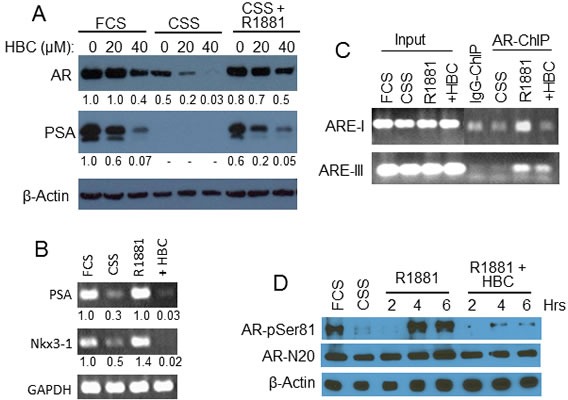
Effect of HBC on androgen-stimulated AR A and B) HBC inhibits androgen-stimulated AR activity. Exponentially growing LNCaP cells (FCS) were hormone-deprived (CSS) and stimulated with 2 nM R1881 in the absence or presence of HBC for 24 hours. AR, PSA and β-actin protein (A) and PSA, NKX3.1, and GAPDH mRNA levels (B) were determined by Western blot and RT-PCR, respectively. Cells treated with 40 μM HBC were used for RT-PCR analysis. Band densities were determined by using the ImageJ program and β-actin- (A) and GAPDH- (B) normalized relative band densities are presented. C) HBC inhibits AR association with PSA- AREs. Hormone-deprived LNCaP cells were treated with 2 nM R1881 in the presence of 40 μM HBC, chromatin-immunoprecipitates were prepared using ant-AR-N20 antibodies (AR-ChIP), and DNA was purified from AR-ChIP and subjected to PCR analysis. ChIP prepared using purified IgG (IgG-ChIP) served as a negative control. Input represents 10% of chromatin used for Immunoprecipitation. D) HBC inhibits phosphorylation of AR Serine 81 residue. Cell extracts prepared from hormone-deprived cells stimulated with R1881 (2 nM) in the presence of HBC (40 μM) for 2, 4, or 6 hours were subjected to Western blot analysis using antibodies against AR-pSer81, AR-N20 and β-actin. All experimental procedures are as described in Materials and Methods. Data in each panel is representative of 2 or more independent experiments.

We next tested the effect of HBC on AR activity stimulated specifically by androgen. As expected, hormone depletion of LNCaP cells, caused by shifting from hormone-rich fetal calf serum (FCS) containing medium to hormone-depleted CSS containing medium, resulted in a dramatic decrease in protein levels of AR and PSA (Fig. 4A). Treatment of cells in CSS medium with HBC further decreased AR protein levels. Addition of R1881 alone to the cells in CSS medium was sufficient to restore AR and PSA to the levels that were in exponentially growing cells in FCS containing medium. However, R1881 treatment in the presence of 20 or 40 μM HBC attenuated the restoration of AR. Importantly, the effect on PSA was greater than that on AR level in HBC treated cells (Fig. 4A), suggesting an inhibitory effect of HBC on AR transcriptional activity. In addition, as seen in exponentially growing cells (Fig. 2C), HBC decreased the expression of cell cycle regulatory proteins, viz., cyclin A, cyclin E, cyclin D1, and Cdk-2, and increased the level of p53 and PARP cleavage in R1881-stimulated cells (Supplementary Fig. S2). Thus, HBC inhibits AR activity and suppresses androgen-stimulated proliferation of PCa cells.

The inhibitory effect of HBC on androgen-stimulated AR activity is also evident from the observation that both PSA and NKX3.1 mRNA levels decreased dramatically when cells in CSS medium were stimulated with R1881 in the presence of HBC; R1881 alone restored the expression of both PSA and NKX3.1 in CSS treated cells to the levels that were in exponentially growing cells in FCS (Fig. 4B). Since *in-vitro* EMSA showed an inhibitory effect of HBC on the binding of AR to PSA-ARE (Fig. 3D), we tested the effect of HBC on *in-vivo* association of AR with PSA-AREs in R1881-stimulated LNCaP cells in CSS medium. PCR analysis of AR-ChIP revealed that R1881 induced the association of AR with ARE-I and ARE-III, and HBC attenuated R1881-induced association of AR with PSA-AREs (Fig. 4C). Thus, HBC suppresses AR activity by blocking androgen-dependent association of AR with AREs.

Since Ser-81 phosphorylation is essential for AR transcriptional activity [24, 25] and is stimulated by androgen [26], we tested the HBC effect on Ser-81 phosphorylation of AR in R1881-stimulated cells. As shown in Fig. 4D, hormone-depletion caused a dramatic decrease in Ser-81 phosphorylation and R1881-induced a robust phosphorylation of AR Ser-81 within 4- to 6-hour treatment of cells in CSS medium, indicating androgen-dependent phosphorylation of AR in LNCaP cells. Importantly, this R1881-induced phosphorylation of AR Ser-81 was completely blocked by HBC, suggesting that the inhibitory effect of HBC on AR activity may involve abrogation of AR Ser-81 phosphorylation.

### HBC effect on global transcriptome in androgen-stimulated PCa cells

**Figure 5:**
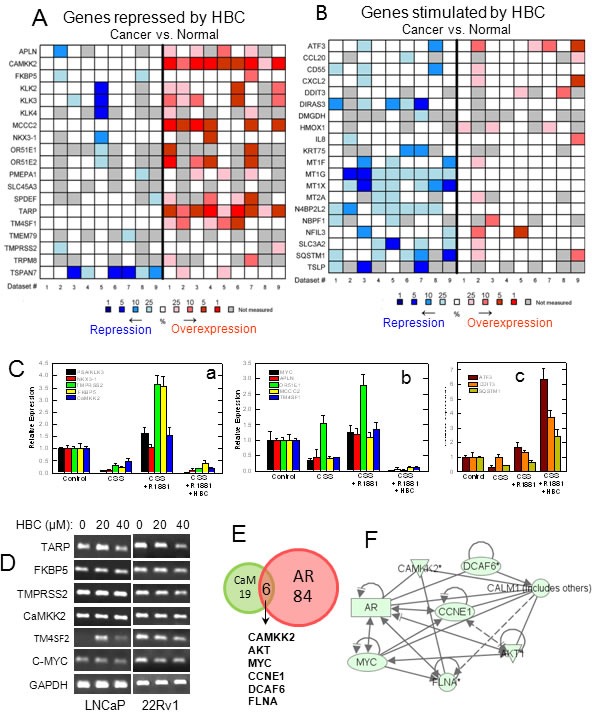
Analysis and validation of HBC affected androgen-regulated gene expression Hormone-deprived LNCaP cells were treated with 2 nM R1881 in the absence or presence of 40 μM HBC for 24 hours and androgen-regulated genes whose expression affected by HBC were identified through RNA-Seq as described in Materials and Methods. A and B) Androgen-regulated genes whose expression was significantly (p<0.002) repressed (A) or stimulated (B) by HBC were analyzed for differential expression in cancer vs. normal prostate tissues by using nine reference data sets (Supplementary Table S2) in Oncomine. The heat maps contain individual studies. The heat map intensity corresponds to percentile overexpression (red) or repression (blue) in prostate carcinoma as compared to normal prostate. C) RT-qPCR validation of representative androgen-regulated genes that were repressed (a and b) or stimulated (c) in HBC treated cells. D) HBC suppresses the expression of androgen/AR-regulated genes in 22Rv1 cells as well as in LNCaP cells. Exponentially growing LNCaP and 22Rv1 cells were treated with HBC (40 μM) for 24 hours, and RT-PCR of selected genes in total RNA was performed as described in Materials and Methods. E) Ingenuity Pathway Analysis (IPA) was used to identify AR- and/or CaM-regulated genes among the genes that were affected significantly (p<0.05) by HBC. F) IPA based identification of network interactions between the eight HBC repressed genes that are regulated both by AR and CaM.

In order to identify HBC effect on androgen-regulated gene expression, we sequenced total RNA isolated from cells that were subjected to androgen-deprivation and then stimulated with R1881 in the absence or presence of HBC. Deep sequencing analysis revealed a significant change (with p<0.05 and 1.5-fold increase or 1.5-fold decrease) in 1,766 transcripts representing 1,475 genes and their isoforms in androgen-deprived cells treated with R1881 in the absence vs. presence of HBC (data not shown). Genes that were altered most significantly (p <0.002) among these were 29 androgen-stimulated genes that were repressed by HBC and 28 androgen-repressed genes that were induced by HBC (Table 1). We analyzed these 57 genes for their expression in cancer vs. normal prostate tissues using the Oncomine Platform. Interestingly, these analyses revealed that the genes that were repressed by HBC are often overexpressed in PCa (Fig. 5A) and, conversely, those that were stimulated by HBC are often down-regulated in PCa (Fig. 5B). This indicates a reversing effect of HBC on the expression of androgen-regulated genes associated with PCa.

**Table 1:** Androgen-regulated genes significantly (p<0.002) affectedby HBC

Androgen-stimulated genes repressed by HBC				Androgen-repressed genes stimulated by HBC		
No.	Gene	P value		No.	Gene	P value
1	KLK3	6.51E-05		1	MT1G	3.98E-05
2	TMPRSS2	0.000165		2	DDIT3	0.000179
3	KLK2	0.00022		3	MT2A	0.000253
4	OR51E1	0.000306		4	MT1F	0.000323
5	SRSF3	0.000401		5	KRT75	0.000333
6	TARP	0.000486		6	SQSTM1	0.000373
7	MYC	0.000521		7	ATF3	0.000437
8	FAM65B	0.000621		8	CD55	0.000626
9	TRPM8	0.000696		9	IL8	0.000681
10	TMEFF2	0.000711		10	HMOX1	0.000736
11	SLC45A3	0.000741		11	CXCL2	0.00083
12	KLK4	0.000842		12	N4BP2L2	0.000895
13	OR51E2	0.000877		13	NFIL3	0.000905
14	NKX3-1	0.000932		14	RAB39B	0.000915
15	TM4SF1	0.000997		15	MT1X	0.000969
16	NRP1	0.001012		16	TSLP	0.001153
17	APLN	0.001057		17	DIRAS3	0.001302
18	PGC	0.001112		18	SERPINB6	0.001312
19	MCCC2	0.001177		19	SLC3A2	0.001372
20	SPDEF	0.001222		20	SEPHS2	0.001402
21	FKBP5	0.001248		21	NEAT1	0.001551
22	PRR15L	0.001298		22	DMGDH	0.00168
23	TMEM79	0.001378		23	CCL20	0.001695
24	TUBA4A	0.001508		24	ATF5	0.001705
25	CAMKK2	0.001528		25	NBPF1	0.001715
26	SRSF2	0.001683		26	ATG4A	0.001725
27	PMEPA1	0.001743		27	AGPAT5	0.001819
28	RRM2	0.001864		28	TRIB3	0.001829
29	MEX3A	0.001984				

The gene expression changes observed in R1881 vs. R1881 + HBC treated cells were validated by RT-qPCR analysis of representative genes (Fig. 5C). Among these genes were some [viz., PSA, NKX3.1, TMPRSS2, FKBP5, and CaMKK2 (Fig. 5Ca)] that are known to be AR-targets and contain AREs, and others that are either overexpressed [viz., MYC, APLN, OR51E<1, MCCC2, and TM4SF1 (Fig. 5Cb)] or down-regulated [viz., ATF3, DDIT3, and SQSTM1 (Fig. 5Cc)] in PCa but are not known to contain AREs, and thus may be indirect targets of AR. In addition, HBC affected the expression of these genes similarly in exponentially growing androgen-sensitive (LNCaP) and castration-resistant (22Rv1) PCa cells (Fig. 5D).

Since HBC inhibits AR activity by binding to CaM, we employed Ingenuity Pathway Analysis (IPA) to identify HBC affected genes that are likely to be regulated by AR and CaM. IPA identified 84 AR-regulated and 19 CaM-regulated genes among the genes that were affected (p<0.05) by HBC (Fig. 5E and Supplementary Table S3). In addition, there were six genes, viz., CaMKK2, AKT, MYC, CCNE1, DCAF6, and FLNA, whose expression known to be regulated both by AR and CaM that were down regulated in HBC treated cells (Fig. 5E). These six gene products are regulated through a direct interaction between them (Fig. 5F). IPA also identified five significant (p values ranging from 2.9×10^−21^ to 8.7×10^−9^) functional categories of genes modulated by HBC (Table 2). These functional categories are relevant to the cell survival, proliferation, and gene expression. Interaction networks between genes that were most significantly (p<0.002) affected by HBC in each of the 5 functional categories is shown in Supplementary Fig. S3. These network analyses indicate that HBC inhibits the expression of genes that promote proliferation and survival, and induces the expression of genes that promote cell death.

**Table 2:** Ingenuity Pathway Analysis of HBC affected androgen-regulated genes

IPA: Molecular and Cellular Functions affected by HBC			
Functional Category	P-value	Number of Differentially Expressed Genes	
		P<0.05	P<0.002
Cell Death and Survival	2.9*10^−21^	215	32
Cellular Development	8.1*10^−15^	202	24
Cellular Growth and Proliferation	4.0*10^−14^	387	24
Cell Cycle	8.0*10^−13^	148	13
Gene Expression	8.7*10^−9^	179	13

Table shows number of genes, whose expression was significantly different between R1881 alone vs. R881 + HBC treated cells, involved in top five functional categories. Pathway enrichment p-values were based on Fisher's exact test.

### HBC inhibits the growth of tumors derived from castration-resistant PCa cells in nude mice

**Figure 6:**
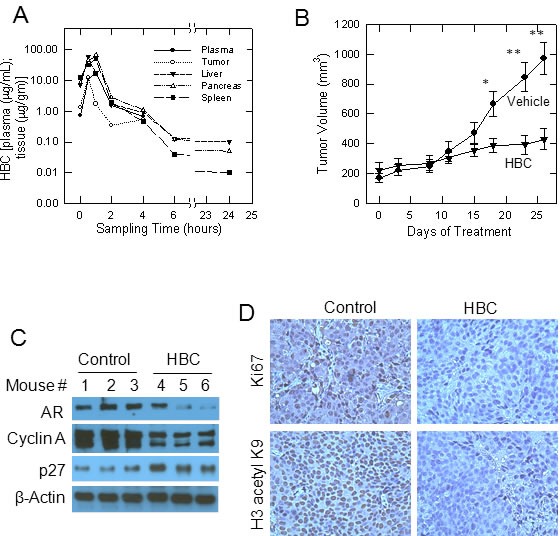
Effect of HBC on tumors derived from castration-resistant C4-2B cells in nude mice A) Concentration-time profiles of HBC in plasma (μg/ml) and in tissues (μg/gm) following intra-peritoneal injection of 100 mg/kg in mice. Each point represents the mean concentration from two mice. B) HBC suppresses the growth of tumors derived from C4-2B cells in nude mice. Nude mice bearing C4-2B tumors were given twice daily (5 days/week) i.p. injection of 100 mg/kg HBC or vehicle and tumor size was measured twice a week as described in Materials and Methods. Data points, mean tumor size (n = 5); bars, SE; *, p<0.05, and **, p<0.01. C) HBC decreases AR and cyclin A levels and increases p27 levels in tumor tissues. Western blot analysis of AR, cyclin A, and β-actin in tissue extracts of tumors from vehicle and HBC treated mice is as described in Materials and Methods. D) HBC suppresses the expression of Ki67 and inhibits histone H3K9 acetylation. Immunostaining of Ki67 and histone H3 acetyl K9 in tumor tissues is as described in Materials and Methods.

Since HBC reversed prostate cancer-associated gene expression (Fig. 5A and 5B), we anticipated an anti-tumorigenic effect of HBC. Therefore, we tested the effect of HBC on the growth of tumors derived from the castration-resistant metastatic PCa cell line C4-2B in nude mice. We first evaluated plasma and tissue distribution of HBC in mice injected with a single dose of 100 mg HBC/kg body weight. Fig. 6A shows the mean concentration-time profiles of HBC in plasma, tumor, liver, pancreas, and spleen following i.p. injection. The plasma and tissue pharmacokinetic parameters are summarized in Supplementary Table S4. Following ip injection, HBC was well absorbed into the systemic circulation; achieving the plasma C_max_ of 16.44 μg/mL at 1 h. HBC exhibited an apparent systemic clearance (CL/F, 5.45 L/kg) similar to the hepatic blood flow in mice (5.4 L/h). HBC was rapidly distributed into tumor tissues (T_max_, 0.5 h) and achieved a tumor drug concentration (i.e., C_max_ and AUC_0-∝_) similar to the systemic (plasma) drug concentration (Supplementary Table S4). Compared to its elimination from the plasma, HBC was eliminated relatively slower from normal and tumor tissues (T_1/2_, 3.3 to 4.7 h) (Fig. 6A and Supplementary Table S4).

Based on the pharmacokinetic data, we treated tumor bearing mice with 100 mg/Kg HBC twice daily/5 days/week. This treatment regimen had no significant effect on either the body weight of mice (Supplementary Fig. S4A) or on gross morphology of cells in major organs such as heart, kidney, liver and lungs (Supplementary Fig. S4B). However, there was a significant decrease (p <0.05) in tumor volume in mice treated with HBC starting 18 days after initiation of treatment as compared to controls. At 23 and 26 days, this difference between tumor volumes in HBC vs. vehicle treated mice was highly significant (p <0.01) (Fig. 6B). The HBC-mediated suppression of tumor growth is due to the suppression of proliferation since the expression of AR and cyclin A required for proliferation were suppressed and the expression of p27, which negatively regulates proliferation, was induced in tumor tissues (Fig. 6C). The anti-proliferative effect of HBC was also evident from a noticeable decrease in Ki67 staining in tumors from HBC treated mice as compared to controls.

Recently, p300 acetyltransferase activity has been reported to be crucial for AR-targeted gene expression [27]. Interestingly, p300 activity is regulated by nitric oxide synthase [22], which is a CaM dependent enzyme [23]. Therefore, we evaluated acetylation of histone H3K9, one of the targets of p300, in prostate tumors from mice treated with HBC. As shown in Fig. 6D, there was a dramatic decrease in H3 acetyl K9 in tumor tissues of mice treated with HBC as compared to controls. Thus the anti-proliferative and anti-tumorigenic effect of HBC may include its inhibitory effect on CaM-modulated p300 acetyltransferase activity required for AR-targeted gene expression.

## DISCUSSION

Current strategies for the treatment of metastatic PCa rely on an intact AR LBD to inhibit AR activity. However, in CRPC, AR LBD is sometimes deleted or mutated rendering AR refractory to all forms of androgen-deprivation and/or AR-antagonists currently available [28, 29]. Therefore, agents targeting AR NTD, which is indispensable for the transcriptional transactivation function of AR, to inhibit AR activity are critical to eliminate the disease. This study identified HBC as one such agent. We have shown previously that CaM binds to the NTD of AR and plays an important role in AR transcriptional activity and AR protein stability [10-12]. In the present study, we showed for first time that CaM is overexpressed in PCa (Fig. 1) and that HBC, which binds exclusively to CaM [20], inhibits AR activity and suppresses the growth of CRPC tumors in nude mice.

We observed a selective inhibitory effect of HBC on proliferation of AR-positive PCa cells (Fig. 2). Since CaM is a ubiquitous protein that regulates many cellular processes [13], the lack of HBC effect on proliferation of AR-negative cells at concentrations that effectively inhibited proliferation of AR-positive PCa cells (Fig. 2) is actually quite striking. It indicates that HBC does not inhibit proliferation simply by inhibiting all CaM-regulated proteins; if it did, proliferation of AR-negative cells should have been similarly inhibited. Since the CaM-binding domains of different CaM–regulated proteins differ significantly in amino acid sequence [30] and structural differences among CaM-binding domains influence CaM affinity for its target [31], it is likely that not all CaM-regulated proteins are inhibited equally by an anti-CaM drug. Indeed, myosin light-chain kinase, protein kinase C, calcineurin, and cAMP phosphodiesterase are all CaM-regulated enzymes, but their sensitivity to trifluoperazine (TFP), a potent anti-CaM drug, varies significantly; TFP inhibits myosin light-chain kinase, protein kinase C, calcineurin, and cAMP phosphodiesterase with an IC_50_ of 140 μM [32], 43.9 μM [33], 20.8 μM [34], and 12.7 μM [33], respectively. In addition, the potency of CaM antagonists may also depend on the intracellular calcium and CaM levels; intracellular calcium levels can affect the binding affinity between CaM and its targets [35], and the sensitivity of CaM-regulated enzymes to anti-CaM drugs. For example, the inhibitory effect of anti-CaM drugs TFP and R24571 on CaM-regulated myosin light-chain kinase activity increases with increasing CaM levels [32]. Therefore, it is possible that CaM overexpression in AR-positive PCa cells (Fig. 1, and [10]) may render CaM-dependent AR activity more sensitive to the inhibitory effect of HBC.

The HBC inhibitory effect on AR activity was associated with the suppression of AR Ser-81 phosphorylation (Fig. 4) and suppression of AR binding to AREs (Figs. 3 and 4), suggesting a role of Ser-81 phosphorylation in AR binding to AREs. This is in agreement with reports that cyclin-dependent kinases, viz., Cdk1 and Cdk9, phosphorylate AR Ser-81 and stabilize AR binding to chromatin [24, 25, 36]. Protein kinases play an important role in regulation of AR function and AR protein stability [37]. Protein kinases activated by growth factors (e.g., EGF and IGF-1) or cytokines (e.g., IL-6 and IL-8) are reported to activate AR in the absence of androgen [38], and several growth factor-stimulated protein kinases including Akt [39], Aurora A [40], Src [41], and cyclin-dependent kinases Cdk1 and Cdk9 [24, 25, 36] that phosphorylate AR depend either directly or indirectly on CaM for kinase activity. For instance, whereas CaM binds directly to and regulates the activities of Akt [42], Aurora A [43], and Src [44] kinases, the activities of Cdk1 and Cdk9 kinases are activated by CaMKII-dependent Cdc25C [45] and CaMK1D [46], respectively. Therefore, it was not surprising that we observed a strong inhibitory effect of HBC on Ser-81 phosphorylation in LNCaP cells (Fig. 4B). These observations raise an intriguing possibility that AR-bound CaM may play a role in the activation of protein kinases that phosphorylate AR and regulate its function and stability. Future studies will test this possibility.

Poor solubility and, therefore, poor bioavailability is a major obstacle when it comes to the usefulness of Curcumin and its derivatives as therapeutic agents [47]. However, although HBC is a Curcumin-derivative, it is more water-soluble than Curcumin since it has additional carboxy and amine groups [20]. Particularly, the sodium salt of HBC (NaHBC/CTK7A) is highly water-soluble and cell permeable [22]. Accordingly, we observed that NaHBC was well absorbed into the systemic circulation of mice within 30 min after i.p. injection of 100 mg/kg. The concentration of HBC was ~25 μM in tumor tissues (C_max_ 12.64 μg/ml) within 1 hour of administration (Fig. 6A and Supplementary Table S4). This concentration is ~5-fold higher than the IC_50_ required for its anti-proliferative effect on LNCaP cells (Fig. 2A), indicating a pharmacologically relevant diffusion of HBC into tumors. This is also evident from the strong inhibitory effect of HBC on the growth of xenografted tumors in mice (Fig. 6B) [22]. The inhibitory effect of HBC on tumor growth is likely due to a cytostatic, rather than cytotoxic, effect since TUNEL assay showed no evidence of apoptosis (data not shown). Cytostatic effect of HBC is evident from a noticeable decrease in cyclin A (Fig. 6C) and Ki67 (Fig. 6D), markers of cell proliferation, and an increase in p27 (Fig. 6C), a negative regulator of cell proliferation. Thus, HBC suppresses tumor growth through an anti-proliferative effect at the concentration (~25 μM) that was observed in tumor tissues (Fig. 6A and Supplementary Table S4). With regards to the safety, it is interesting to note that despite high HBC levels in liver (58 μg/gm at 30 min), pancreas (68 μg/gm at 1 hour), and spleen (52 μg/gm at 1 hour) (Fig. 6A and Table S5), there were no noticeable changes in gross morphology (Supplementary Fig. S4B), suggesting that cells in these normal tissues are insensitive to HBC. The safety of HBC is also evident from the observation that there was no significant change in the body weight of mice treated with 100 mg/kg twice daily/5 days/week for 4 weeks (Supplementary Fig. S4A) [22].

In summary, these studies demonstrate for the first time that HBC, which binds exclusively to CaM, inhibits AR activity and suppresses the proliferation of both androgen-sensitive and castration-resistant AR-positive prostate cancer cells. In a xenograft model, HBC inhibits the growth of CRPC tumors. Thus, these studies establish CaM as a viable target to inhibit AR activity through an AR LBD-independent mechanism and provide proof-of-principle for safe and effective usefulness of HBC as a therapeutic agent to inhibit the growth of CRPC.

## MATERIALS AND METHODS

### Antibodies and reagents

Antibodies against AR-N20, cyclin A, cyclin E, cyclin D1, Cdk2, PCNA, p53, p27, p21, PARP1, Ki67, PSA, and actin were all from Santa Cruz Biotechnology (Dallas, TX), cyclin B from BD Biosiences, (San Jose, CA), AR-pSer81 and calmodulin from EMD Millipore (Billerica, MA), and H3 (acetyl K9) from Abcam (Cambridge, MA). Bicalutamide (Casodex) was from LKT Laboratories (St. Paul, MN) and synthetic androgen methyltrienolone (R1881) was from Perkin Elmer (Waltham, MA). HBC was provided by one of the investigator (HJK). Sodium salt of HBC (NaHBC/CTK7A) was purchased from EMD Millipore (Billerica, MA) and also provided by one of the investigators (TKK). While HBC was used in studies presented in Figs. 2A, 2B, 3A and 3B, all other studies were carried out using NaHBC/CTK7A.

### Cell lines and cell culture

LNCaP, 22Rv1, PC-3, DU145, and NIH3T3 cell lines were purchased from American Type Culture Collection (ATCC, Monassas, VA), C4-2B cells were from Dr. Fazlul H. Sarkar (Wayne State University School of Medicine, Detroit, MI) and BPH-1 cells from Dr. Simon W. Hayward (Vanderbilt University Medical Center, Nashville, TN). LNCaP, C4-2B, 22Rv1 and BPH-1 cells in RPMI-1640 medium (Life Technologies, Grand Island, NY), and PC-3, DU145 and NIH3T3 cells in DMEM medium (Life Technologies) were supplemented with 10% fetal bovine serum (Atlanta Biologicals, Flowery Branch, GA), 2.5 mM glutamine, 100 μg/ml streptomycin, and 100 units/ml penicillin, and grown in a humidified incubator with 5% CO_2_ and 95% air at 37^O^C. The medium for C4-2B cells also contained 10 mM HEPES.

Experiments with LNCaP cells were carried out when they were at <15 passages from the time of receiving from ATCC. Cell proliferation was monitored by pulse labeling with ^3^H-thymidine for 1 hour as described previously [10] or by cell count using a Coulter Counter (Zf Model, Coulter Electronics, Inc., Hialeah, FL). For hormone deprivation, exponentially growing LNCaP cells at a density of 50-70% confluence were shifted into phenol red-free RPMI medium (Life Technologies) supplemented with 6% charcoal-stripped fetal calf serum (CSS) (Atlanta Biologicals), 2.5 mM glutamine, 100 μg/ml streptomycin, and 100 units/ml penicillin for 36 hours. In order to test the effect of HBC on androgen-stimulated events, hormone-deprived cells were then treated with 2 nM synthetic androgen R1881 in the absence or presence of HBC.

### Immunoprecipitation and Western blotting

Cell extracts prepared as described previously [11] were diluted 10-fold in 250 mM NaCl and 50 mM Tris (pH 7.4) and incubated overnight with 4 μg/ml of anti-AR-N20 or anti-CaM antibody at 4^O^C. The immune complexes were then adsorbed to protein A/G agarose immunoadsorbent (Pierce, Rockfold, IL) by incubation for 1 hour at ambient temperature with gentle agitation. The adsorbed complexes were washed twice in dilution buffer by centrifugation at 4^O^C and eluted with PAGE loading buffer (Bio-Rad, Richmond, CA). Control immunoprecipitates were prepared by using 4 μg/ml purified rabbit or mouse IgG (Antibodies Incorporated, Davis, CA) in place of anti-AR or anti-CaM antibodies. Western blotting of the samples dissolved in PAGE loading buffer were carried out as described previously [11].

### RNA isolation, RT-PCR and quantitative PCR analysis

Total RNA was extracted from cells with RNeasy Mini Kit (Qiagen, Valencia, CA) according to the manufacturer's protocols and RT-PCR was performed as described previously [48]. Quantitative PCR (qPCR) was carried out using SYBR Select Master Mix (Applied Biosystems, Life Technologies) on an Applied Biosystems 7500 Fast Real-Time PCR System (Applied Biosystems, Foster City, CA). The cycle number at which the reaction crossed a threshold fluorescence (C_T_) was determined for each transcript, and the level of expression of each test gene relative to GAPDH (reference) mRNA was determined using the equation 2^−ΔCT^, where ΔC_T_ = C_T, test_ – C_T, reference_ [49]. The primers used for PCR and qPCR were as in Supplementary Table S1.

### Chromatin-immunoprecipitation (ChIP) and PCR analysis of androgen response elements (AREs)

Preparation of soluble chromatin from LNCaP cells treated without (control) or with HBC, and the immunoprecipitation of chromatin using antibodies against AR-N20 or purified IgG (control) was as described by Jia et al [50]. DNA purified from ChIP samples was subjected to PCR analysis using the following PSA-AREs primers: 5′-tctgcctttgtcccctagat-3′ (forward) and 5′-aaccttcattccccaggact-3′ (reverse) for PSA ARE-I, and 5′-gcctggatctgagagagatatcatc-3′ (forward) and 5′-acacctttttttttctggattgttg-3′ (reverse) for PSA ARE-III. Procedures for PCR reaction and visualization of amplified products were as described previously [48].

### Gel electrophoretic mobility shift assay (EMSA)

Soluble nuclear extracts were prepared from exponentially growing LNCaP and DU145 cells and EMSA was performed using DIG High Prime DNA Labeling and Detection Starter Kit II (Roche Applied Science, Indianapolis, IN). The sequences of PSA-ARE probes used in EMSA were: 5′-tgcagaacagcaagtgctagc-3′ (forward) and 5′-gctagcacttgctgttctgca-3′ (reverse). Digoxygenin (DIG)-labeled probes were incubated with 5 μg nuclear extract in a binding buffer containing 24 mM HEPES, pH 7.9, 8 mM Tris HCL, pH 8.0, 12% glycerol, 2 mM EDTA, 1 mM dithiotreitol, 3 μg BSA, and 1 μg poly [d(I-C)] in a final volume of 20 μl for 30 min on ice. Samples were then subjected to 6% native polyacrylamide gel electrophoreses, transferred onto positively charged nylon membrane (Roche Applied Science), and PSA-ARE probe signal on nylon membrane was then detected using an anti-DIG-alkaline phosphatase conjugate and chemiluminescence.

### RNA-Seq and data analysis

Total RNA prepared as described above was submitted to Applied Genomics Technology Center, Wayne State University School of Medicine, where RNA integrity was confirmed using Agilent 2000 TapeStation system, sequencing adaptor ligated cDNA libraries were constructed using TruSeq RNA Sample Preparation Kit v2 (Illumina, San Diego, CA), and 50 cycle paired-end sequencing was done on Illumina HiSeq 2500. Details of RNA-Seq data analysis can be found in Supplementary Materials and Methods.

### Oncomine Analysis

A set of 57 genes (Table 1), whose expression was significantly (p<0.002) affected by HBC, were assessed for differential expression in cancer vs. normal prostate tissues by using nine reference data sets in the Oncomine Platform (Oncomine v4.5, Life Technologies, Ann Arbor, MI). Each of the reference data sets had an expression profile of more than 8500 genes in at least 54 prostate samples (Supplementary Table S2).

### Ingenuity Pathway Analysis

Functional interactions between the set of genes whose expression was either decreased or increased significantly (1,475 gene set with p<0.05, and 57 gene set with p<0.002) in cells treated with R1881 + HBC, as compared to R1881 alone, was evaluated using the Ingenuity curated knowledge base as a reference and Ingenuity Pathway Analysis (IPA) tool (Ingenuity Systems, Redwood City, CA).

### Human prostate tumor xenograft mouse model

The animal protocols were reviewed and approved by the Institutional Laboratory Animal Care and Use Committee of Henry Ford Hospital. Subcutaneous (s.c.) injection of C4-2B cells (5 × 10^6^) and measurement of the volume of tumors derived from C4-2B cells in six week old male athymic nude mice was as described previously [51]. When xenografts reached a volume of 175 ± 58 mm^3^, the tumor-bearing mice were randomly assigned to a solvent-treated (control) or HBC-treated group and given either solvent (100 μl PBS) or HBC intra-peritoneally (i.p.) 100 mg/kg twice daily for 5 consecutive days/week. The experiment was terminated when the control tumors reached ~1,100 mm^3^ (on day 28) and the mice were sacrificed. Part of each tumor was used to prepare the tissue extracts and the remainder was fixed and paraffin embedded. Tissue extracts for Western blotting were prepared as described previously [51].

### Pharmacokinetics and tissue distribution of HBC

The pharmacokinetics and tissue distributions of HBC were examined in tumor-bearing mice weighing 25.7 ± 0.6 gms. The mice were given a single dose of 100 mg/kg HBC i.p and then blood, tumors, and major organs (i.e., liver, pancreas, and spleen) were collected at 5 min, and 0.5, 1, 2, 4, 6, and 24 hours, with two mice for each time point. A detailed description of procedures for measuring HBC levels in plasma and tissue samples and pharmacokinetic data analysis can be found in Supplementary Materials and Methods.

### Immunostaining

Benign and tumor human prostate tissue sections were obtained with approval from the Institutional Review Board of Henry Ford Health System. Tissue sections were subjected to heat-induced epitope retrieval using DAKO PT LINK Chamber and immunostained on the DAKO LINK Autostainer using EnVision FLEX reagents (DAKO, Carpinteria, CA) by following the manufacturer's suggested protocol. Primary antibodies used for staining were rabbit monoclonal antibodies against CaM (Abcam) diluted 1:3,500, and mouse monoclonal antibodies against AR (DAKO) diluted 1:75. Immunostaining of mouse tissue sections with antibodies against Ki67 and H3 (acetyl K9) was performed manually using reagents from Vector Laboratories (Burlingame, CA) by following the manufacturer's suggested protocol.

## SUPPLEMENTARY MATERIALS FIGURES AND TABLES



## References

[1] Siegel R, Naishadham D, Jemal A (2013). Cancer statistics, 2013. CA Cancer J Clin.

[2] Schiewer MJ, Augello MA, Knudsen KE (2012). The AR dependent cell cycle: mechanisms and cancer relevance. Molecular and cellular endocrinology.

[3] Feldman BJ, Feldman D (2001). The development of androgen-independent prostate cancer. Nat Rev Cancer.

[4] Bastos DA, Dzik C, Rathkopf D, Scher HI (2014). Expanding androgen- and androgen receptor signaling-directed therapies for castration-resistant prostate cancer. Oncology (Williston Park, NY).

[5] Augello MA, Den RB, Knudsen KE (2014). AR function in promoting metastatic prostate cancer. Cancer metastasis reviews.

[6] Mostaghel EA, Plymate SR, Montgomery B (2014). Molecular pathways: targeting resistance in the androgen receptor for therapeutic benefit. Clin Cancer Res.

[7] Sadar MD (2011). Small molecule inhibitors targeting the “achilles' heel” of androgen receptor activity. Cancer Res.

[8] Dehm SM, Tindall DJ (2007). Androgen receptor structural and functional elements: role and regulation in prostate cancer. Mol Endocrinol.

[9] Heemers HV, Tindall DJ (2007). Androgen receptor (AR) coregulators: a diversity of functions converging on and regulating the AR transcriptional complex. Endocr Rev.

[10] Cifuentes E, Mataraza JM, Yoshida BA, Menon M, Sacks DB, Barrack ER, Reddy GP (2004). Physical and functional interaction of androgen receptor with calmodulin in prostate cancer cells. Proc Natl Acad Sci U S A.

[11] Pelley RP, Chinnakannu K, Murthy S, Strickland FM, Menon M, Dou QP, Barrack ER, Reddy GP (2006). Calmodulin-androgen receptor (AR) interaction: calcium-dependent, calpain-mediated breakdown of AR in LNCaP prostate cancer cells. Cancer Res.

[12] Sivanandam A, Murthy S, Chinnakannu K, Bai VU, Kim SH, Barrack ER, Menon M, Reddy GP (2011). Calmodulin protects androgen receptor from calpain-mediated breakdown in prostate cancer cells. J Cell Physiol.

[13] Berchtold MW, Villalobo A (2014). The many faces of calmodulin in cell proliferation, programmed cell death, autophagy, and cancer. Biochim Biophys Acta.

[14] Chafouleas JG, Pardue RL, Brinkley BR, Dedman JR, Means AR (1981). Regulation of intracellular levels of calmodulin and tubulin in normal and transformed cells. Proc Natl Acad Sci U S A.

[15] Liu GX, Sheng HF, Wu S (1996). A study on the levels of calmodulin and DNA in human lung cancer cells. Br J Cancer.

[16] MacManus JP, Braceland BM, Rixon RH, Whitfield JF, Morris HP (1981). An increase in calmodulin during growth of normal and cancerous liver *in vivo*. FEBS Lett.

[17] Krishnaraju K, Murugesan K, Vij U, Kapur BM, Farooq A (1991). Calmodulin levels in oestrogen receptor positive and negative human breast tumours. Br J Cancer.

[18] Li L, Sacks DB (2007). Functional interactions between calmodulin and estrogen receptor-alpha. Cellular signalling.

[19] Gallo D, Jacquot Y, Laurent G, Leclercq G (2008). Calmodulin, a regulatory partner of the estrogen receptor alpha in breast cancer cells. Molecular and cellular endocrinology.

[20] Shim JS, Lee J, Park HJ, Park SJ, Kwon HJ (2004). A new curcumin derivative, HBC, interferes with the cell cycle progression of colon cancer cells via antagonization of the Ca2+/calmodulin function. Chemistry & biology.

[21] Jung HJ, Kim JH, Shim JS, Kwon HJ (2010). A novel Ca2+/calmodulin antagonist HBC inhibits angiogenesis and down-regulates hypoxia-inducible factor. J Biol Chem.

[22] Arif M, Vedamurthy BM, Choudhari R, Ostwal YB, Mantelingu K, Kodaganur GS, Kundu TK (2010). Nitric oxide-mediated histone hyperacetylation in oral cancer: target for a water-soluble HAT inhibitor, CTK7A. Chemistry & biology.

[23] Bredt DS, Snyder SH (1990). Isolation of nitric oxide synthetase, a calmodulin-requiring enzyme. Proc Natl Acad Sci U S A.

[24] Chen S, Gulla S, Cai C, Balk SP (2012). Androgen receptor serine 81 phosphorylation mediates chromatin binding and transcriptional activation. J Biol Chem.

[25] Gordon V, Bhadel S, Wunderlich W, Zhang J, Ficarro SB, Mollah SA, Shabanowitz J, Hunt DF, Xenarios I, Hahn WC, Conaway M, Carey MF, Gioeli D (2010). CDK9 regulates AR promoter selectivity and cell growth through serine 81 phosphorylation. Mol Endocrinol.

[65%] Gioeli D, Ficarro SB, Kwiek JJ, Aaronson D, Hancock M, Catling AD, White FM, Christian RE, Settlage RE, Shabanowitz J, Hunt DF, Weber MJ (2002). Androgen receptor phosphorylation. Regulation and identification of the phosphorylation sites. J Biol Chem.

[27] Zhong J, Ding L, Bohrer LR, Pan Y, Liu P, Zhang J, Sebo TJ, Karnes RJ, Tindall DJ, van Deursen J, Huang H (2014). p300 acetyltransferase regulates androgen receptor degradation and PTEN-deficient prostate tumorigenesis. Cancer Res.

[28] Yuan X, Cai C, Chen S, Chen S, Yu Z, Balk SP (2014). Androgen receptor functions in castration-resistant prostate cancer and mechanisms of resistance to new agents targeting the androgen axis. Oncogene.

[29] Schmidt LJ, Tindall DJ (2013). Androgen receptor: past, present and future. Current drug targets.

[30] Rhoads AR, Friedberg F (1997). Sequence motifs for calmodulin recognition. Faseb J.

[31] Crivici A, Ikura M (1995). Molecular and structural basis of target recognition by calmodulin. Annual review of biophysics and biomolecular structure.

[32] Zimmer M, Hofmann F (1984). Calmodulin antagonists inhibit activity of myosin light-chain kinase independent of calmodulin. Eur J Biochem.

[33] Norman JA, Ansell J, Stone GA, Wennogle LP, Wasley JW (1987). CGS 9343B, a novel, potent, and selective inhibitor of calmodulin activity. Mol Pharmacol.

[34] Gong CX, Shaikh S, Grundke-Iqbal I, Iqbal K (1996). Inhibition of protein phosphatase-2B (calcineurin) activity towards Alzheimer abnormally phosphorylated tau by neuroleptics. Brain Res.

[35] Chin D, Means AR (2000). Calmodulin: a prototypical calcium sensor. Trends Cell Biol.

[36] Chen S, Xu Y, Yuan X, Bubley GJ, Balk SP (2006). Androgen receptor phosphorylation and stabilization in prostate cancer by cyclin-dependent kinase 1. Proc Natl Acad Sci U S A.

[37] Koryakina Y, Ta HQ, Gioeli D (2014). Androgen receptor phosphorylation: biological context and functional consequences. Endocr Relat Cancer.

[38] Wang G, Sadar MD (2006). Amino-terminus domain of the androgen receptor as a molecular target to prevent the hormonal progression of prostate cancer. J Cell Biochem.

[39] Taneja SS, Ha S, Swenson NK, Huang HY, Lee P, Melamed J, Shapiro E, Garabedian MJ, Logan SK (2005). Cell-specific regulation of androgen receptor phosphorylation *in vivo*. J Biol Chem.

[40] Shu SK, Liu Q, Coppola D, Cheng JQ (2010). Phosphorylation and activation of androgen receptor by Aurora-A. J Biol Chem.

[41] Guo Z, Dai B, Jiang T, Xu K, Xie Y, Kim O, Nesheiwat I, Kong X, Melamed J, Handratta VD, Njar VC, Brodie AM, Yu LR, Veenstra TD, Chen H, Qiu Y (2006). Regulation of androgen receptor activity by tyrosine phosphorylation. Cancer cell.

[42] Coticchia CM, Revankar CM, Deb TB, Dickson RB, Johnson MD (2009). Calmodulin modulates Akt activity in human breast cancer cell lines. Breast Cancer Res Treat.

[43] Plotnikova OV, Pugacheva EN, Dunbrack RL, Golemis EA (2010). Rapid calcium-dependent activation of Aurora-A kinase. Nature communications.

[44] Hayashi N, Nakagawa C, Ito Y, Takasaki A, Jinbo Y, Yamakawa Y, Titani K, Hashimoto K, Izumi Y, Matsushima N (2004). Myristoylation-regulated direct interaction between calcium-bound calmodulin and N-terminal region of pp60v-src. J Mol Biol.

[45] Patel R, Holt M, Philipova R, Moss S, Schulman H, Hidaka H, Whitaker M (1999). Calcium/calmodulin-dependent phosphorylation and activation of human Cdc25-C at the G2/M phase transition in HeLa cells. J Biol Chem.

[46] Ramakrishnan R, Rice AP (2012). Cdk9 T-loop phosphorylation is regulated by the calcium signaling pathway. J Cell Physiol.

[47] Anand P, Kunnumakkara AB, Newman RA, Aggarwal BB (2007). Bioavailability of curcumin: problems and promises. Mol Pharm.

[48] Bai VU, Kaseb A, Tejwani S, Divine GW, Barrack ER, Menon M, Pardee AB, Reddy GP (2007). Identification of prostate cancer mRNA markers by averaged differential expression and their detection in biopsies, blood, and urine. Proc Natl Acad Sci U S A.

[49] Livak KJ, Schmittgen TD (2001). Analysis of relative gene expression data using real-time quantitative PCR and the 2(−Delta Delta C(T)) Method. Methods (San Diego, Calif.

[50] Jia L, Kim J, Shen H, Clark PE, Tilley WD, Coetzee GA (2003). Androgen receptor activity at the prostate specific antigen locus: steroidal and non-steroidal mechanisms. Molecular cancer research : MCR.

[51] Kaseb AO, Chinnakannu K, Chen D, Sivanandam A, Tejwani S, Menon M, Dou QP, Reddy GP (2007). Androgen receptor and E2F-1 targeted thymoquinone therapy for hormone-refractory prostate cancer. Cancer Res.

