# Supplementary material for: Hydrazinobenzoylcurcumin inhibits androgen receptor activity and growth of castration-resistant prostate cancer in mice
Source: Oncotarget. 2015 Jan 31;6(8):6136–50. doi: 10.18632/oncotarget.3346 (PMC4467427; doi:10.18632/oncotarget.3346)
Supplement: Supplementary file 1 [file oncotarget-06-6136-s001.pdf]

Hydrazinobenzoylcurcumin inhibits androgen receptor activity and growth of castration-resistant prostate cancer in mice

Supplementary Materials and Methods

Pharmacokinetics and tissue distribution of HBC:

RNA-Seq data analysis: The output for the RNA-Seq data used for the statistical analysis was the reads per kilobase per million mapped reads (RPKM). To ensure that the selected transcripts represent substantive changes, we selected only those where the enriched sample had a greater expression than the hormone-deprived cell sample. Since the distribution of RPKM was skewed, the analysis used the log of the [RPKM measurement plus 1] as the analysis metric. The test statistic employed was the difference in RPKM between the R1881-stimulated (R1881) sample and the R1881-stimulated in the presence of HBC (R1881 + HBC) sample, which because of the log transformation is equivalent to a log-fold change. A multiple testing correction was employed to minimize the false discovery rate (FDR). Assuming that only 2% of the transcripts are differentially expressed, we computed the nominal statistical threshold (0.0018) to achieve 10% FDR. A permutation technique was employed to determine the transcripts that were differentially expressed between the 2 conditions. Briefly, we shuffled the labels of the 4 samples measured (viz., exponentially growing (control), hormone-deprived (CSS treated), R1881-stimulated, and R1881 + HBC treated) creating a mock androgen-deprived, R1881-stimulated and R1881+ HBC sample to generate log-fold change values following the same algorithm used for the actual data analysis. All possible permutations were done to create a null distribution for log-fold change. A p-value was then computed for each transcript by comparing the observed log-fold change to the permutation-generated null distribution.

Measurement of HBC levels in plasma and tissues: The blood samples were centrifuged at 3000 rpm, at 4^0C for 10 min, and plasma was collected immediately after the centrifugation and stored at -80^0C until analysis. The tissue samples were washed free of blood with 0.9% saline, blotted dry, weighed, and stored at -80^0C until analysis. HBC in mouse plasma and tissue samples were determined

using a high-performance liquid chromatography coupled with tandem mass spectrometry (LC-MS/MS) method as described below. The tissue samples were homogenized in 4 volumes of human plasma. A 250 μl aliquot of tissue homogenate or mouse plasma was spiked with 1 ml ethyl acetate. The mixture was vortex-mixed, centrifuged at 14,000 rpm for 5 min, and the top layer was transferred and evaporated to dryness under a stream of nitrogen in a water bath at 50^0C ± 5^0C. The residue was reconstituted in 100 μl of methanol/water containing 0.45% formic acid (70:30, v/v), and centrifuged at 14,000 rpm for 5 min. Ten microliters of the supernatant was injected into the LC-MS/MS. The chromatographic separation was achieved on a Waters XTerra MS column (2.1 × 50 mm, 3.5 μm i.d.) with a mobile phase consisting of methanol/water containing 0.45% formic acid (70:30, v/v) at a flow rate of 0.2 ml/min. The column effluent was monitored using a Waters Quattro Micro^{TM} triple quadruple mass-spectrometric detector equipped with electrospray ionization source (Milford, MA, USA). HBC was monitored in the positive ionization mode at the mass transition of 485.3→469.4. The calibration curve was constructed in human plasma over the concentration range of 2 to 10,000 ng/ml. The within- and between-day precision and accuracy of the assay were <15%.

Pharmacokinetic data analysis: The plasma and tissue pharmacokinetic parameters of HBC were estimated using noncompartmental analysis with the WinNonlin software (Pharsight). The maximum concentration (C_{max}) and the time of occurrence for maximum concentration (T_{max}) were obtained by visual inspection of the plasma/tissue concentration-time curves after the drug administration. The total area under the concentration-time curve from time zero to the last measurable time point (AUC_{last}) was calculated using the linear and logarithmic trapezoidal method for the ascending and descending plasma concentrations, respectively. The total area under the concentration-time curve from time zero to infinity ($AUC_{0-\infty}$) was calculated as the sum of AUC_{last} and the extrapolated area, which was calculated by the last observed concentration divided by the terminal rate constant (λ_z), where λ_z was estimated by terminal log-linear phase of the concentration-time curve. The terminal elimination half-life

($T_{1/2,}$) was calculated as $0.693/\lambda_z$. The apparent systemic clearance (CL/F) was calculated as Dose/$AUC_{0-\infty}$. The apparent volume of distribution (V_z/F) was estimated as (CL/F)/λ_z.

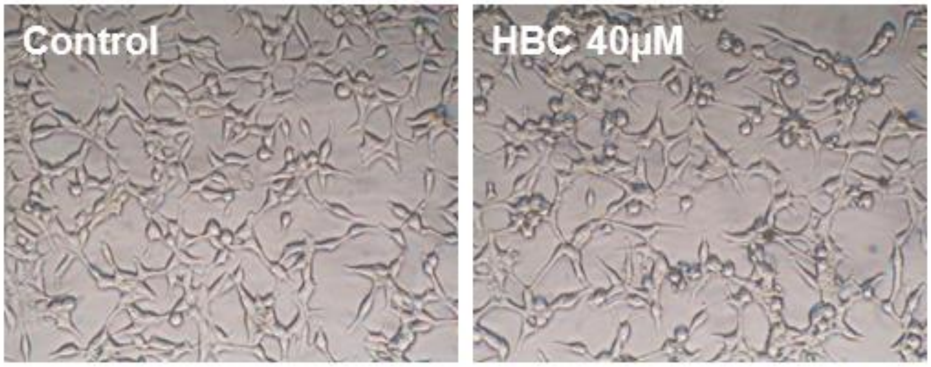

Figure S1: Effect of HBC on morphology of LNCaP cells: Exponentially growing LNCaP cells were treated with vehicle (0.01% DMSO, control) or 40 μM HBC for 24 hours and phase contrast images were captured using Zeiss Invertoscope 40C microscope.

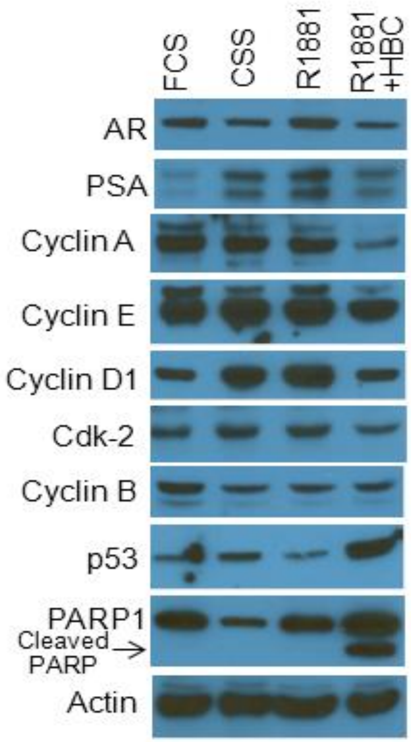

Figure S2: Effect of HBC on androgen-regulated expression of cell cycle proteins in LNCaP cells. Exponentially growing (FCS) LNCaP cells deprived for androgen (CSS) were treated with R1881 (2 nM) in the absence (R1881) or presence of NaHBC (40 µM) (R1881+HBC) for 24 hours. Cell extracts were prepared and Western blotting performed as described in Materials and Methods.

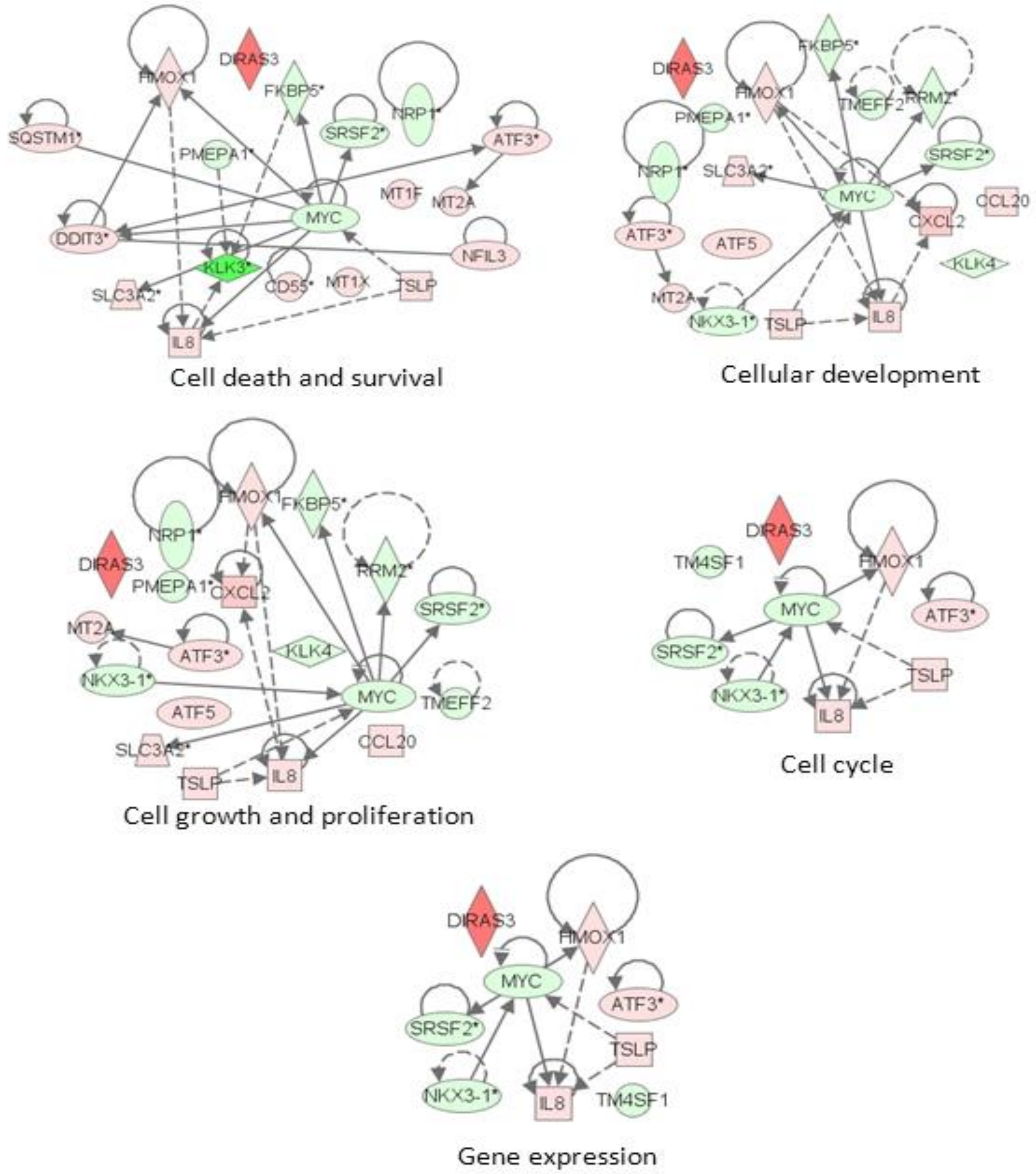

Figure S3: Identification of network interactions between HBC affected genes. IPA based biological interaction network between HBC affected genes (p<0.002) within the five functional categories in Table 2.

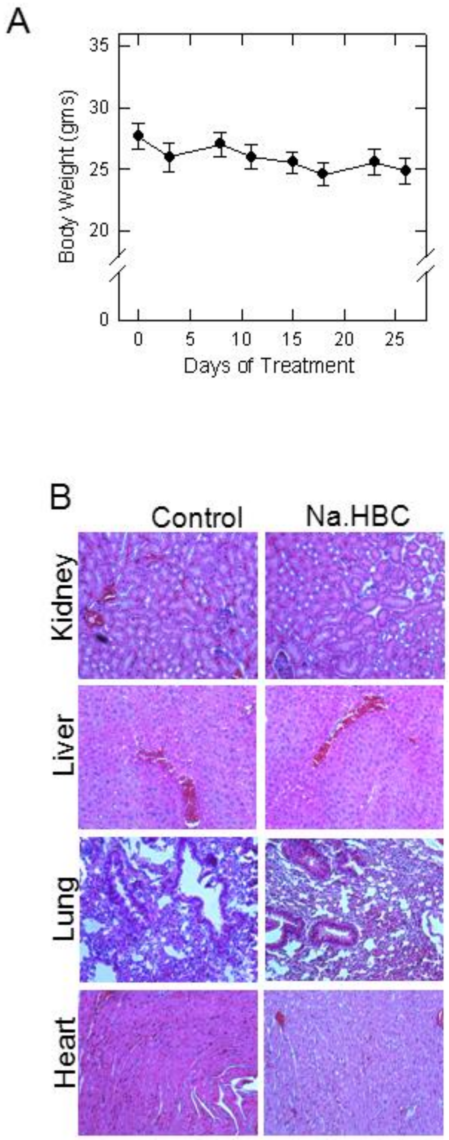

Figure S4: A) Body weight of mice is unaffected by HBC. Nude mice bearing C4-2B tumors were given a daily (5 days/week) i.p. injection of 100 mg/Kg and body weight was measured twice a week. Data points, mean body weight (n=5); bars, SE. B) HBC has no noticeable effect on histology of major organs, viz., kidney, liver, lung and heart. Paraffin-embedded tissue sections were deparaffinized, hydrated, and stained with hematoxylin for 1 min. After rinsing, the slides were stained with eosin for 1 min, rinsed thoroughly, and mounted with Permount.

Table S1: PCR primer Sequences

Genes	Primer Sequences	
	**Forward**	**Reverse**
PSA	5’-gcacccggagagctgtgt-3’	5’-gatcacgcttttgttcctgat-3’
NKX3-1	5’-ccgcagaagcgctcccgagct-3’	5’-cgaggagagctgctttcgctt-3’
CAMKK2	5’-gcagtgacgcgctcctctccaa-3’	5’-tccgctcgtccatgaatgggca-3’
FKBP5	5’-aaatccaaacgaaggagcaa-3’	5’-gccacatctctgcagtcaaa-3’
TMPRSS2	5’-ccatttgcaggatccgtctg-3’	5’-ggatgtgtcttggggagcaa-3’
MYC	5’-tgaggagacaccgcccac-3’	5’-caacatcgatttcttcctc-3’
APLN	5’- ccagagggtcaaggaatgggc-3’	5’- ataaccgccgggggtgggca-3’
OR51E1	5’-acacattccttccatacggttga-3’	5’-tagcactggattcattgccatt-3’
MCCC2	5’-gctcgtatctcagtgatgggag-3’	5’- aaagccgcttcatcagcactgg-3’
TM4SF1	5’-agcaccgagggccagtacctt-3’	5’-ccaccaagagccaagaggatag-3’
TM4SF2	5’-aagtgtatgttgggagtgtttcgagt-3’	5’-gagctttgaccccatctatcagc-3’
ATF3	5’-aggatgctctgctgtttcct-3’	5’-gacaaagggcgtcaggttag-3’
DDIT3	5'-ccttcagtgacttccatgac-3'	5'-tcaccatacagcagcctgag-3'
SQSTM1	5’-atcggaggatccgagtgt-3’	5’-tggctgtgagctgctctt-3’
GAPDH	5’-gagatccctccaaaatcaagtg-3’	5’-ccttccacgataccaaagttgt-3’

Table S2

References of datasets* used for Oncomine analysis

**#**	**Study**<br>**Samples**	**Genes**	
1	Prostate Carcinoma vs. Normal (Grasso Prostate)	19189	122
2	Prostate Carcinoma vs. Normal (Holzbeierlein Prostate)	8603	54
3	Prostate Carcinoma vs. Normal (Lapointe Prostate)	10166	112
4	Prostate Carcinoma vs. Normal (Liu Prostate)	12624	57
5	Prostate Carcinoma vs. Normal (Liu Prostate 2)	18823	74
6	Prostate Carcinoma vs. Normal (Singh Prostate)	8603	102
7	Prostate Carcinoma vs. Normal (Taylor Prostate 3)	22238	185
8	Prostate Carcinoma Epithelia vs. Normal (Tomlins Prostate)	10656	101
9	Prostate Carcinoma vs. Normal (Yu Prostate)	8603	112

*, See Oncomine for details on each of the references

Table S3: AR- and CaM-regulated genes affected by HBC

1. **AR regulated:**

Gene symbol	Fold Change	p-value	Location	Family	Entrez Gene ID
ABCC4	-3.43524	0.005301	Plasma Membrane	transporter	10257
AMD1	-3.07523	0.006688	Cytoplasm	enzyme	262
AR	-2.0181	0.040947	Nucleus	ligand-dependent nuclear receptor	367
ATAD2	-1.97381	0.034138	Nucleus	other	29028
ATF3	27.28272	0.001173	Nucleus	transcription regulator	467
AURKA	3.156553	0.049307	Nucleus	kinase	6790
AZGP1	-4.70213	0.0073	Extracellular Space	transporter	563
CALM3	-1.93919	0.029509	Cytoplasm	other	801|805|808
CAST	13.11909	0.002411	Cytoplasm	peptidase	831
CBX1	4.312353	0.031093	Nucleus	transcription regulator	10951
CCND1	-3.88995	0.004729	Nucleus	other	595
CDKN1A	6.142206	0.008664	Nucleus	kinase	1026
CEBPB	9.455696	0.002247	Nucleus	transcription regulator	1051
DCTPP1	-2.0218	0.026864	Cytoplasm	enzyme	79077
DHCR24	-2.05032	0.023632	Cytoplasm	enzyme	1718
E2F1	-2.12171	0.033803	Nucleus	transcription regulator	1869
ERBB2	-2.16612	0.029309	Plasma Membrane	kinase	2064
ERBB3	-2.17684	0.021308	Plasma Membrane	kinase	2065
ETV1	-4.4852	0.008121	Nucleus	transcription regulator	2115
ETV5	28.28571	0.008033	Nucleus	transcription regulator	2119
FAM174B	-1.76938	0.04499	unknown	other	400451
FEN1	-1.96053	0.036072	Nucleus	enzyme	2237
FKBP5	-9.83154	0.048427	Nucleus	enzyme	2289
GDF15	3.950328	0.010906	Extracellular Space	growth factor	9518
HMGB2	2.968137	0.029806	Nucleus	transcription regulator	3148
HSPA5	7.059296	0.002515	Cytoplasm	enzyme	3309
IGF1R	-3.15068	0.018056	Plasma Membrane	transmembrane receptor	3480

IGFBP3	4.085242	0.03593	Extracellular Space	other	3486
IL1R1	-2.07322	0.037355	Plasma Membrane	transmembrane receptor	3554
JMJD1C	2.896672	0.038943	Nucleus	other	221037
KAT2B	7.284722	0.007536	Nucleus	transcription regulator	8850
KLK2	-56.9825	0.00257	Extracellular Space	peptidase	3817
KLK3	-74.0917	0.001232	Extracellular Space	peptidase	354
KRT14	-33.5	0.022701	Cytoplasm	other	3861
LMNB1	-1.74796	0.048602	Nucleus	other	4001
MAOA	-2.04466	0.027495	Cytoplasm	enzyme	4128
MCM2	-2.68073	0.023272	Nucleus	enzyme	4171
MCM4	-2.45869	0.020616	Nucleus	enzyme	4173
MDM2	4.315856	0.009778	Nucleus	transcription regulator	4193
MME	-3.0324	0.009679	Plasma Membrane	peptidase	4311
MRAS	6.021782	0.037789	Plasma Membrane	enzyme	22808
MSMB	-10.6667	0.013061	Extracellular Space	other	4477
MTA2	-1.7182	0.049068	Nucleus	transcription regulator	9219
NCOR1	6.776781	0.036596	Nucleus	transcription regulator	9611
NDRG1	-3.16775	0.007776	Nucleus	kinase	10397
NKX3-1	-6.74093	0.000932	Nucleus	transcription regulator	4824
PA2G4	-1.79202	0.041533	Nucleus	transcription regulator	5036
PARK7	-1.75168	0.043412	Nucleus	enzyme	11315
PGC	-34.2683	0.001112	Extracellular Space	peptidase	5225
PIK3R1	-4.3684	0.010015	Cytoplasm	kinase	5295
PMEPA1	-14.8923	0.001743	Plasma Membrane	other	56937
PRKCD	-2.97236	0.0101	Cytoplasm	kinase	5580
REPS2	-4.61923	0.037039	Cytoplasm	other	9185
RHOB	-3.60805	0.004875	Cytoplasm	enzyme	388
RNF6	3.528591	0.04032	Nucleus	transcription regulator	6049
RRM2	-6.91357	0.04745	Nucleus	enzyme	6241
RUVBL1	-1.7284	0.046779	Nucleus	transcription regulator	8607
SETD7	2.721632	0.039718	Nucleus	enzyme	80854
SHBG	557406.7	0.016459	Extracellular	other	6462

			Space		
SHC1	-5.12115	0.00264	Cytoplasm	kinase	6464
SIRT1	3.87951	0.026515	Nucleus	transcription regulator	23411
SLC45A3	-8.76129	0.000741	Cytoplasm	other	85414
SMAD1	5.112759	0.006875	Nucleus	transcription regulator	4086
SMARCA4	-2.08567	0.036198	Nucleus	transcription regulator	6597
SNAI2	-2.70769	0.022235	Nucleus	transcription regulator	6591
SPDEF	-11.006	0.006618	Nucleus	transcription regulator	25803
SREBF1	-2.17593	0.026894	Nucleus	transcription regulator	6720
SVIL	3.610888	0.025774	unknown	other	6840
TACC2	2.999196	0.030835	Nucleus	other	10579
TARP	-20.1111	0.000486	Cytoplasm	other	445347
TGIF1	-2.8032	0.03252	Nucleus	transcription regulator	7050
TH1L	-1.90953	0.033151	Nucleus	other	51497
TMPRSS2	-17.539	0.000165	Plasma Membrane	peptidase	7113
TNFAIP2	-9.35714	0.049139	Extracellular Space	other	7127
TPD52	-2.17987	0.021393	Cytoplasm	other	7163
TRIM68	-3.3842	0.005767	Cytoplasm	enzyme	55128
TUBB3	-3.05966	0.014534	Cytoplasm	other	10381
UBE2L3	-3.56751	0.043262	Nucleus	enzyme	7332
VEGFA	6.80086	0.031879	Extracellular Space	growth factor	7422
VIM	-1.85232	0.035892	Cytoplasm	other	7431
ZBTB1	10.35934	0.046812	Nucleus	other	22890
ZBTB16	-2.74396	0.016483	Nucleus	transcription regulator	7704
ZMIZ1	-2.2314	0.025867	Nucleus	other	57178
ZWINT	22.1916	0.005359	Nucleus	other	11130

2. CaM regulated genes:

Gene symbol	Fold Change	p-value	Location	Family	Entrez Gene ID
VIPR1	-7.62432	0.011869	Plasma Membrane	G-protein coupled receptor	7433
AURKB	-7.38378	0.01764	Nucleus	kinase	9212
GRB7	-7.05055	0.024123	Plasma Membrane	other	2886

KCNN2	-3.40592	0.011498	Plasma Membrane	ion channel	3781
RAB3B	-3.12983	0.009183	Cytoplasm	enzyme	5865
CALD1	-3.01892	0.01989	Cytoplasm	other	800
SYNE2	-2.82813	0.025406	Nucleus	other	23224
GRK1	-2.5443	0.039053	Plasma Membrane	kinase	6011
RDX	2.731957	0.045793	Cytoplasm	other	5962
NOL7	2.789189	0.04202	Nucleus	other	51406
INVS	3.468545	0.037059	Nucleus	transcription regulator	27130
GCLM	3.680431	0.017602	Cytoplasm	enzyme	2730
DDX21	5.358563	0.009728	Nucleus	enzyme	9188
HMMR	5.565636	0.014272	Plasma Membrane	transmembrane receptor	3161
LYST	5.578199	0.030452	Cytoplasm	transporter	1130
OPTN	7.290033	0.03855	Cytoplasm	other	10133
DOCK3	7.913043	0.006969	Cytoplasm	other	1795
SQSTM1	21.29912	0.000373	Cytoplasm	transcription regulator	8878

3. Both AR and CaM Regulated:

Gene symbol	Fold Change	p-value	Location	Family	Entrez Gene ID
MYC	-10.5381	0.000521	Nucleus	transcription regulator	4609
CAMKK2	-6.44435	0.001528	Cytoplasm	kinase	10645
FLNA	-2.82055	0.010411	Cytoplasm	other	2316
AKT1	-2.58702	0.018873	Cytoplasm	kinase	207
DCAF6	-3.0237	0.031518	Nucleus	transcription regulator	55827
CCNE1	-2.27632	0.032305	Nucleus	transcription regulator	898

Table S4: Plasma and tissue pharmacokinetic parameters[a] of HBC

	**Plasma**	**Tumor**	**Liver**	**Pancreas**	**Spleen**
T_{max} (h)	1.00	0.50	0.50	1.00	1.00
C_{max} (µg/mL for plasma, µg/g for tissues)	16.44	12.64	58.11	68.28	52.19
AUC_{last} (h*µg/mL for plasma, h*µg/g for Tissues	18.34	10.35	42.97	66.69	49.06
$AUC_{0-\infty}$ (h*µg/mL for plasma, h*µg/g for Tissues	18.34	10.40	43.48	67.05	49.09
$T_{1/2}$ (h)	0.48	3.50	3.44	4.69	3.33
CL/F (L/h/kg)	5.45	n/a	n/a	n/a	n/a
Vz/F (L/kg)	3.76	n/a	n/a	n/a	n/a

[a] Pharmacokinetic parameters were estimated using noncompartmental analysis of the mean concentration time profiles from two mice.

n/a, not estimated
